# The impact of preoperative atrial fibrillation on survival and outcomes in heartmate II and heartmate 3 patients

**DOI:** 10.1186/s12872-025-04680-1

**Published:** 2025-03-29

**Authors:** Rami Al-khusein, Alish Kolashov, Nikolaus Marx, Ajay Moza, Ahmad Abugameh, Yousif Ibrahim Mohamed, Assila Al-Ahmed, Mohammed Shoaib, Leyla Dogan, Rashad Zayat, Mohammad Amen Khattab

**Affiliations:** 1 Faculty of Medicine, Clinic for Cardiology, Angiology, and Intensive Care Medicine, RWTH Aachen University, University Hospital Aachen, Aachen, Germany; 2 Faculty of Medicine, Department of Cardiac Surgery, RWTH Aachen University, University Hospital Aachen, Aachen, Germany; 3 Heart Center Trier, Department of Cardiovascular and Thoracic surgery, Barmherzige Brueder Hospital, Trier, Germany; 4 Department of Cardiovascular Surgery, Klinikum Dortmund gGmbH, Dortmund, Germany; 5 Witten/Herdecke University, Witten, Germany; 6 Department of Cardiothoracic Surgery, St. Johannes Hospital, Dortmund, Germany; 7 Department of Radiology, St. Augustinus Hospital, Dueren, Germany; 8 Department of Cardiac Surgery, Klinikum Links der Weser, Bremen, Germany

**Keywords:** Left ventricular assist device, Atrial fibrillation, HeartMate 3, HeartMate II, Ischemic stroke, Survival

## Abstract

**Background:**

Atrial fibrillation (AF) is prevalent among patients with left ventricular assist devices (LVADs); however, the exact influence of different types of AF on the clinical outcomes of these patients is unknown. The purpose of this study was to ascertain the impact of different types of AF on the outcomes of patients with LVADs.

**Methods:**

The records of 162 patients with the LVADs HeartMate 3 (*n* = 64) and HeartMate II (*n* = 98) at a single center were reviewed. Kaplan‒Meier survival analysis and Cox proportional hazards regression were used to analyze the associations among paroxysmal atrial fibrillation (pAF), permanent atrial fibrillation (peAF), survival, hemorrhage, and thromboembolism. To adjust for confounding factors, we also used the inverse probability of treatment weighting (IPTW) method and estimated the average treatment effect (ATE) for the outcomes of ischemic stroke (IS), mortality and right heart failure (RHF). Clinical trial number: not applicable.

**Results:**

Of the 56 (34.6%) patients with preoperative AF, 38 patients had pAF and 18 patients had peAF. Overall survival differed significantly among the three groups (log-rank test: *p* = 0.001). Patients with peAF or pAF had a greater incidence of postoperative right heart failure (RHF) than did patients with sinus arrhythmia (SR) (*p* = 0.037). According to the univariate and multivariate Cox regression analyses, both pAF and peAF were independent risk factors for post-LVAD IS (hazard ratio [HR]: 3.65 (95% CI: 1.28–10.41, *p* = 0.016 and HR: 9.48 (2.94–30.56), *p* < 0.001). Patients with preoperative peAF or pAF who received an LVAD did not have an increased risk for hemorrhagic stroke. After IPTW, the ATE with respect to mortality was significantly different between patients with peAF and those with SR (*p* < 0.001). The ATE with respect to post-LVAD IS was significantly different for the pAF vs. SR and the peAF vs. SR patient groups (*p* = 0.035 and *p* = 0.004). After IPTW, the ATE for the RHF outcome did not significantly differ among the three groups.

**Conclusions:**

Patients with preoperative pAF or peAF who underwent LVAD implantation had a higher risk of IS than patients with SR. Our data demonstrated that only preoperative peAF is associated with poor survival while SR is not.

## Background

Left ventricular assist devices (LVADs) for patients with end-stage heart failure (HF) are now recognized as an acceptable treatment modality [1, 2]. Despite the notable enhancements in survival rates, functional capabilities, and quality of life that LVADs offer, their implementation frequently coincides with severe adverse events such as cerebrovascular accidents (CVAs), pump thrombosis (PT), and major infections [3, 4].

As many as 50% of HF patients have atrial fibrillation (AF) [5], which has been linked to poorer outcomes and higher mortality in these patients [6]. The occurrence of AF or atrial flutter in patients with LVAD is significant, as documented incidence rates range from 21 to 72% [7, 8]. Multiple studies have presented findings on the outcomes of patients with preoperative AF who have LVADs compared with those of individuals without AF. However, these studies have yielded inconsistent results [7, 8, 9, 10].

Therefore, the objective of this study was to analyze the effects of different types of preoperative AF (paroxysmal AF (pAF) and permanent AF (peAF)) on the survival rate and occurrence of adverse events in HeartMate II (HMII) and Heartmate 3 (HM3) patients.

## Methods

We retrospectively reviewed the medical records of adult patients who underwent HMII or HM3 implantation in our department between January 2012 and December 2021.

The clinical course and information regarding LVAD implantation were obtained from our institutional database. In addition to demographic and clinical data, we examined our database for preoperative cardiac rhythm, preoperative and postoperative laboratory data, and the incidence of adverse events, including bleeding and thromboembolism.

Our retrospective data analysis received approval from the ethics committee of our institution (Ethikkommission-RWTH Aachen, IRBP 10/2014, EK151/09-Version-1.3). Due to the retrospective nature of our study, informed consent was waived by our institutional ethics board.

### AF and anticoagulation

To assess the occurrence of pre- and postoperative atrial fibrillation (AF), retrospective chart evaluations, which included apparatus interrogations, progress notes, and electrocardiogram findings, were conducted. AF can be categorized into two subtypes, paroxysmal atrial fibrillation (pAF) and permanent atrial fibrillation (peAF), according to established definitions [5]. Due to potential variations in results between these categories, the patients were categorized into three groups for analysis: (1) individuals with sinus arrhythmia (SR); (2) individuals with pAF; and (3) individuals with peAF. The HF specialist was given the authority to determine how to treat AF after LVAD implantation. All patients were administered a daily dose of 100 mg of aspirin and warfarin for anticoagulation. The target international normalized ratio (INR) range for HMII patients without AF was 2.5–3.0, while for HM3 patients, the range was 2.0–2.5. The INR range for all patients with atrial fibrillation (AF) was set at 2.5–3.0 for both HMII and HM3 devices. If a patient with AF experienced numerous instances of bleeding, the target INR was reduced to a range of 1.8–2.0.

### AF and outcomes

This study aimed to investigate the effects of pAF and peAF on three primary outcomes: survival, thromboembolic events, and bleeding. All patient deaths were verified by reviewing the medical records, and the specific cause of death was documented. Major bleeding, PT and right heart failure (RHF) were defined based on the updated 2021 version of the Thoracic Surgeon Interagency Registry of Mechanically Assisted Circulatory Support (STS-Intermacs) [11].

### Statistical analysis

Categorical variables are presented as absolute numbers and percentages. The chi-square test or Fisher’s exact test was used to examine categorical variables. The Kolmogorov‒Smirnov test was used to test for a normal distribution. The t test or Mann‒Whitney U test was used to evaluate continuous data where appropriate. Normally distributed continuous variables are presented as the means ± standard deviations. Nonnormally distributed variables are represented as medians with interquartile ranges (IQRs). Kaplan‒Meier time-to-event curves were generated for death and ischemic stroke (IS) and were stratified by AF state. The statistical significance between the curves was assessed via the log-rank test, after which we used the Bonferroni method to adjust for multiple comparisons among the three groups. A linear regression analysis was performed to detect whether pAF or peAF had an effect on postoperative right heart failure (RHF). Cox proportional hazards univariable and multivariable regression analyses were conducted to examine the impact of preoperative AF and other known risk factors for AF on postoperative outcomes.

Known risk factors with a *p* value of < 0.05 in the univariable analysis were included in the multivariable model. To adjust for observed confounding factors in our observational study, we used the inverse probability of treatment weighting (IPTW) method, which has two primary stages. Initially, the probability—or propensity—of exposure to the relevant risk factor or intervention is determined on the basis of an individual’s attributes (i.e., propensity score). Then, weights are determined as the reciprocal of the propensity score. The use of these weights for the research population generates a pseudo population whose confounders are uniformly distributed between exposed and unexposed groups [12]. Due to its resemblance to randomized controlled trials, inverse probability of treatment weighting (IPTW) is extensively employed in comparative effectiveness research [12]. After performing IPTW, we estimated the average treatment effect (ATE) in our population using SR, pAF and peAF as treatments after adjusting for age, sex, preoperative kidney disease, diabetes mellitus, hypertension, and ischemic cardiomyopathy (ICM). We estimated the average treatment effect in our population via the abovementioned method using post-LVAD ischemic stroke (IS), mortality, and post-LVAD RHF as outcomes.

All *p* values were 2-tailed, and the level of significance for all *p* values was < 0.05. All analyses were conducted via R statistics in R Studio interface version 4.0.3 (RStudio Team, Boston, MA) in conjunction with the jamovi project (2020) (jamovi computer software, version 2.3.9; JAMovi.org), which may be accessed at https://www.jamovi.org/.

## Results

A total of 162 patients (19.1% were female) diagnosed with end-stage heart failure underwent LVAD implantation and received either the HM3 (*n* = 64) or the HMII (*n* = 98) device at our institution during the study period. A total of 73.5% of the patients experienced ischemic cardiomyopathy (ICM), while 26.5% had dilated cardiomyopathy (DCM). The mean follow-up time was 24.4 ± 20.5 months. Preoperatively, sinus arrhythmia (SR) was documented in 106 patients, and AF was documented in 56 patients. Thirty-eight (23.4%) patients had pAF and 18 (11.1%) patients had peAF before implantation. In all, 13.2% of the patients in the SR group, 23.7% in the pAF group and 44.4% in the peAF group were female (*p* = 0.006). Table 1 displays the patients’ preoperative clinical and demographic information. Patients in the pAF and peAF groups were significantly older than those in the SR group (63.6 ± 8.9 years vs. 64.3 ± 7.0 years vs. 60.1 ± 9.4 years, *p* = 0.045). Patients in the SR group had a significantly greater incidence of ICM as a cause of heart failure than did patients in the pAF and peAF groups (*p* = 0.007). However, patients with pAF or peAF had a significantly greater incidence of DCM (*p* = 0.012) (Table 1). Patients with pAF or peAF also had significantly greater incidences of preoperative kidney disease (KD) than did patients with SR (42.1% vs. 55.6% vs. 27.4%, *p* = 0.031) and had higher levels of preoperative creatinine (SR: 1.2 ± 0.9 mg/dL vs. pAF: 1.6 ± 0.7 mg/dL vs. peAF: 1.5 ± 0.5 mg/dL, *p* = 0.049). Preoperatively, left atrium appendage (LAA) thrombus was detected through transesophageal echocardiography in 3 patients in the pAF group and in 2 patients in the peAF group among the remaining patients in the SR group (*p* = 0.003). The distribution of the performed operative procedures did not differ among the three groups (Table 2); only the percentage of patients who underwent LVAD implantation plus LAA closure was significantly greater in the pAF and peAF groups because the LAA thrombus was detected preoperatively in these patients (SR: 0 vs. pAF: 7.9% vs. 11.1%, *p* = 0.006).

**Table 1:** Patient demographics and preoperative data

	SR (*n* = 106)	pAF (*n* = 38)		peAF (*n* = 18)		*P*	
Age (years)	60.1 ± 9.4	63.6 ± 8.9		64.3 ± 7.0		**0.045**	
Female n (%)	14 (13.2)	9 (23.7)		8 (44.4)		**0.006**	
BMI kg/m^2^	27.4 ± 4.6	27.9 ± 4.4		27.5 ± 4.2		0.855	
HeartMate 3 n (%)	33 (31.1)	21 (55.3)		10 (55.6)		**0.011**	
HeartMate II n (%)	73 (68.9)	17 (44.7)		8 (44.4)		**0.011**	
ICM n (%)	85 (80.2)	21 (55.3)		11 (61.1)		**0.007**	
DCM n (%)	19 (17.9)	15 (39.5)		7 (38.9)		**0.012**	
EF (%)	19.4 ± 5.7	20.1 ± 4.6		20.2 ± 5.0		0.682	
IDDM n (%)	13 (12.3)	10 (26.3)		5 (27.8)		0.066	
PAD n (%)	24 (22.6)	10 (26.3)		7 (38.9)		0.337	
CVD n (%)	24 (22.6)	3 (7.9)		2 (11.1)		0.092	
Nicotine n (%)	64 (60.4)	20 (52.6)		9 (50.0)		0.565	
AHT n (%)	76 (71.7)	28 (73.7)		17 (94.4)		0.120	
Preop IS n (%)	3 (2.8)	3 (7.9)		2 (11.1)		0.205	
Hyperlipidemia n (%)	48 (45.3)	19 (50.0)		7 (38.9)		0.731	
KD n (%)	29 (27.4)	16 (42.1)		10 (55.6)		**0.031**	
Preop dialysis n (%)	7 (6.7)	2 (5.3)		0 (0.0)		0.521	
COPD n (%)	30 (28.3)	13 (34.2)		4 (22.2)		0.629	
PH n (%)	64 (60.4)	23 (60.5)		13 (72.2)		0.624	
Beta-Blocker n (%)	66 (62.3)	25 (65.8)		13 (72.2)		0.698	
Digitalis n (%)	13 (12.3)	15 (39.5)		10 (55.6)		**< 0.001**	
Amiodarone n (%)	17 (16.0)	30 (78.9)		4 (22.2)		**< 0.001**	
Prior eCV n (%)	0 (0.0)	16 (42.1)		8 (44.4)		**< 0.001**	
LAA thrombus	0 (0.0)	3 (7.9)		2 (11.1)		**0.006**	
Prior cardiac surgery n (%)	12 (16.4)	5 (29.4)		0 (0.0)		0.178	
Prior PCI n (%)		36 (34.0)	16 (42.1)	10 (55.6)		0.188	
Detected thrombus on LAA		0	4 (10.5)	4 (22.2)		< 0.001	
INTERMACS profile n (%)							
I	13 (12.3)	4 (10.5)		1 (5.6)		0.101	
II	15 (14.2)	5 (13.2)		2 (11.1)			
III	15 (14.2)	11 (28.9)		8 (44.4)			
IV	63 (59.4)	18 (47.4)		7 (38.9)			
**Preoperative laboratory data**:							
LDH U/L	228 (198, 303)	241 (202, 315)		226 (211, 298)		0.898	
AST U/L	31.0 (22.0, 48.5)	28.5 (22.0, 52.0)		26.0 (19.2, 54.0)		0.729	
ALT U/L	34.5 (20.0, 66.2)	30.5 (25.2, 53.2)		23.5 (17.8, 35.5)		0.159	
Hb mg/dL	12.4 ± 2.4	12.0 ± 2.4		12.2 ± 1.6		0.765	
Platelet counts G/L	226 (183, 288)	199 (141, 277)		211 (185, 312)		0.065	
INR	1.1 ± 0.2	2.1 ± 0.2		2.3 ± 0.2		**< 0.001**	
NT-pro-BNP pg/mL	2496 (1417, 7863)	2411(1925, 8251)		2900 (2900, 6064)		0.715	
Creatinine mg/dL	1.2 ± 0.9	1.6 ± 0.7		1.5 ± 0.5		**0.049**	

Continuous data are presented as the mean ± SD or as the median (25th, 75th percentiles) where appropriate. ALT: alanine aminotransferase; AST: aspartate aminotransferase; BMI: body mass index; BSA: body surface area; COPD: chronic obstructive pulmonary disease; CVD: cerebrovascular disease; DCM: dilated cardiomyopathy; DM: diabetes mellitus; eCV: electric cardioversion; EF: ejection fraction; Hb: hemoglobin; ICM: ischemic cardiomyopathy; ICU: intensive care unit; INR: international normalized ratio; INTERMACS: Interagency Registry for Mechanically Assisted Circulatory Support; KD: kidney disease; LAA: left atrium appendage; LDH: lactate dehydrogenase; NT-pro-BNP: N-terminal pro-brain natriuretic peptide; PAD: peripheral arterial disease; PCI: percutaneous coronary intervention; PH: pulmonary hypertension; pAF: paroxysmal atrial fibrillation; peAF: permanent atrial fibrillation; SR: sinus arrhythmia. Bold text indicates a significant *p* value

**Table 2:** Perioperative data and postoperative course

	SR *n* = 106	pAF *n* = 38	peAF *n* = 18	*P*
LVAD alone n (%)	67 (63.2)	24 (63.2)	9 (50.0)	0.555
LVAD + LAA closure	0 (0.0)	3 (7.9)	2 (11.1)	**0.006**
LVAD + CABG n (%)	33 (31.4)	8 (21.1)	3 (16.7)	0.263
LVAD + TVR n (%)	4 (3.8)	2 (5.3)	3 (16.7)	0.087
LVAD + AVR n (%)	2 (2.3)	1 (4.8)	1 (10.0)	0.412
Pneumonia n (%)	44 (41.5)	24 (63.2)	9 (50.0)	0.070
Sepsis n (%)	41 (38.7)	21 (55.3)	6 (33.3)	0.151
ICU stay (days)	13.9 ± 17.1	23.4 ± 23.1	15.8 ± 14.1	**0.026**
Postoperative RHF	8 (7.5)	5 (13.2)	5 (27.8)	**0.037**
Postop KD n (%)	33 (31.1)	21 (55.3)	13 (72.2)	**0.001**
Postop ischemic stroke n (%)	7 (6.6)	7 (18.4)	5 (27.7)	**0.012**
Hemorrhagic stroke n (%)	1 (0.9)	1 (2.6)	1 (5.6)	0.374
TIA n (%)	9 (8.5)	0 (0.0)	0 (0.0)	0.228
Hemothorax n (%)	13 (12.3)	12 (31.6)	5 (27.8)	**0.018**
GI-Bleeding n (%)	7 (6.6)	2 (5.3)	0 (0.0)	0.525
LVAD thrombus n (%)	8 (7.5)	0 (0.0)	2 (11.1)	0.165
**Laboratory data before discharge**:				
LDH U/L	356 (296, 453.)	313.5 (276.5, 383)	411 (325.5, 549)	0.056
AST U/L	29 (22, 40.2)	30 (22.5, 36.8)	41(33, 47.2)	0.067
ALT U/L	30 (19.5 to 57)	26 (20.5 to 32.8)	22 (17.2, 57.5)	0.614
INR	2.2 ± 0.3	2.8 ± 0.3	2.9 ± 0.2	**< 0.001**
Hb g/dL	9.5 ± 1.0	9.7 ± 1.0	9.1 ± 0.9	0.114

Continuous data are presented as the mean ± SD or as the median (25th, 75th percentiles) where appropriate. ALT: alanine aminotransferase; AST: aspartate aminotransferase; CABG: coronary artery bypass graft; GI-bleeding: gastrointestinal bleeding; Hb: hemoglobin; ICU: intensive care unit; INR: international normalized ratio; LAA: left atrium appendage; LDH: lactate dehydrogenase; LVAD: left ventricular assist device; TVR: tricuspid valve replacement

### Atrial fibrillation and clinical outcomes

**Fig. 1:**
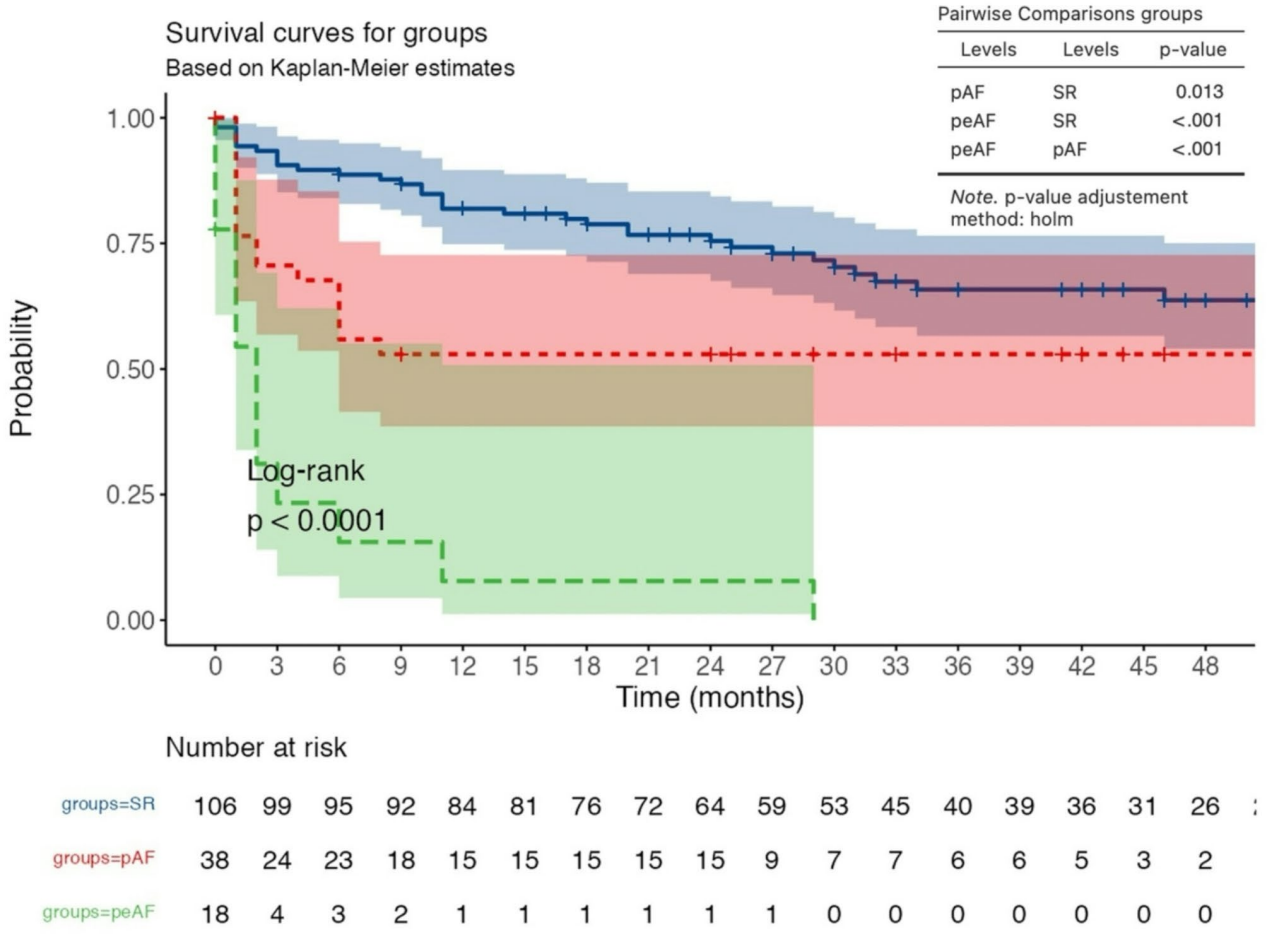
Kaplan‒Meier survival curves pAF: paroxysmal atrial fibrillation; peAF: permanent atrial fibrillation; SR: sinus arrhythmia

The mean survival time was 31.2 ± 19.5 months in the SR group, 15.8 ± 17.6 months in the pAF group and 3.2 ± 7.1 months in the peAF group. Overall survival differed significantly among the three groups (log-rank test: *p* < 0.001) (Fig. 1). When we compared the pAF group with the SR group, we found that patients with pAF had a significantly lower probability of survival (log-rank: *p* = 0.031), and when we compared the peAF group with the SR group, we observed a significantly lower probability of survival in those with peAF (log-rank: *p* = 0.001). Table 2 summarizes the postoperative outcomes of all patients. The incidence of postoperative pneumonia and sepsis did not differ among the groups (Table 2). Patients with preoperative pAF or peAF had a greater incidence of postoperative RHF than did patients with SR (SR: 7.5% vs. pAF: 13.2% vs. peAF: 27.8%, *p* = 0.037). Patients with preoperative pAF or peAF also had longer ICU stays than patients with preoperative SR (pAF: 23.4 ± 23.1 days vs. peAF: 15.8 ± 14.1 days vs. SR: 13.9 ± 17.1 days, *p* = 0.026). A total of 31.1% of patients with SR, 55.3% of patients with pAF and 72.2% of patients with peAF had postoperative KD (*p* = 0.001). The incidence of postoperative ischemic stroke (IS) was significantly greater in both the pAF and peAF groups than in the SR group (18.4% vs. 27.7% vs. 6.6%, *p* = 0.012). The frequency of postoperative hemorrhagic stroke (HS) did not differ among the three groups (Table 2). The incidence of pump thrombosis also did not differ among the three groups. Patients with preoperative pAF or peAF had a greater incidence of rethoracotomy than did those in the SR group (31.6% vs. 27.8% vs. 12.3%, *p* = 0.018). At hospital discharge, all laboratory data were similar among the three groups, despite the different INR values, which were significantly greater in the pAF and peAF groups than in the SR group (2.8 ± 0.3 vs. 2.9 ± 0.2 vs. 2.2 ± 0.3, *p* < 0.001).

**Fig. 2:**
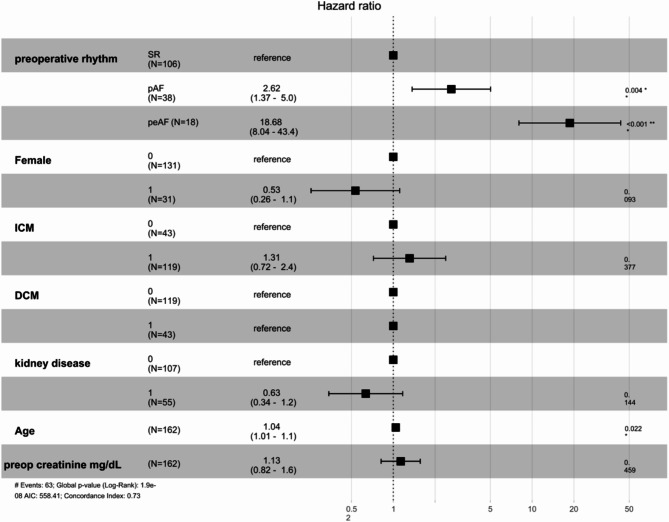
Multivariate Cox regression hazard ratio plots for predicting mortality DCM: dilated cardiomyopathy; pAF: paroxysmal atrial fibrillation; peAF: permanent atrial fibrillation; SR: sinus arrhythmia

After entering age, sex, ICM, DCM, preoperative KD, preoperative creatinine and the preoperative AF status in the univariate and multivariate Cox regression analyses to explore whether the preoperative AF type is an independent risk factor for post-LVAD mortality (Table 3), we found a significantly greater association between pAF and an increased risk of mortality than between SR and increased mortality risk (hazard ratio [HR]: 2.62 (1.37–5.02, *p* = 0.004)) (Fig. 2; Table 3). Patients with preoperative peAF who underwent LVAD implantation also had an increased risk of mortality (HR: 18.68 (8.04–43.40, *p* < 0.001)) (Fig. 2; Table 3). Preoperative pAF was an independent risk factor for postoperative IS according to univariate and multivariate Cox regression analyses (HR: 4.35 (1.40–13.51, *p* = 0.011)) (Table 4). Similarly, compared with SR, preoperative peAF was also an independent risk factor for postoperative IS (HR: 14.95 (3.94–56.68, *p* < 0.001)) (Table 4). We then performed a linear regression analysis with RHF as a dependent factor, and after entering age, sex, ICM, DCM, preoperative KD, preoperative creatinine and the preoperative AF status, we found that peAF was an independent risk factor for postoperative RHF (odds ratio: 11.2 (95% CI: 0.72–12.4), *p* = 0.003) (Table 5).

**Table 3:** Univariable and multivariable Cox regression for predicting mortality after LVAD implantation

		All	HR (univariable)	HR (multivariable)
Preoperative rhythm	SR	106 (65.4)	-	-
	pAF	38 (23.5)	2.16 (1.18–3.94, *p* = 0.012)	2.62 (1.37–5.02, *p* = 0.004)
	peAF	18 (11.1)	10.42 (5.35–20.26, *p* < 0.001)	18.68 (8.04–43.40, *p* < 0.001)
Females	0	131 (80.9)	-	-
	1	31 (19.1)	0.71 (0.35–1.44, *p* = 0.342)	0.53 (0.26–1.11, *p* = 0.093)
ICM	0	43 (26.5)	-	-
	1	119 (73.5)	0.72 (0.42–1.23, *p* = 0.230)	1.31 (0.72–2.38, *p* = 0.377)
DCM	0	119 (73.5)	-	-
	1	43 (26.5)	1.38 (0.81–2.35, *p* = 0.230)	NA (NA-NA, *p* = NA)
Kidney disease	0	107 (66.0)	-	-
	1	55 (34.0)	1.29 (0.78–2.15, *p* = 0.326)	0.63 (0.34–1.17, *p* = 0.144)
Age	Mean (SD)	61.4 (9.2)	1.03 (1.00-1.07, *p* = 0.029)	1.04 (1.01–1.08, *p* = 0.022)
Preop creatinine mg/dL	Mean (SD)	1.4 (0.8)	1.21 (0.98–1.50, *p* = 0.074)	1.13 (0.82–1.56, *p* = 0.459)

SR: sinus arrhythmia; pAF: paroxysmal atrial fibrillation; peAF: permanent atrial fibrillation; ICM: ischemic cardiomyopathy; DCM: dilated cardiomyopathy

**Table 4:** Univariable and multivariable Cox regression for predicting ischemic stroke after LVAD implantation

		All	HR (univariable)	HR (multivariable)
Preoperative rhythm	SR	106 (65.4)	-	-
	pAF	38 (23.5)	3.65 (1.28–10.41, *p* = 0.016)	4.35 (1.40-13.51, *p* = 0.011)
	peAF	18 (11.1)	9.48 (2.94–30.56, *p* < 0.001)	14.95 (3.94–56.68, *p* < 0.001)
Female	0	131 (80.9)	-	-
	1	31 (19.1)	0.76 (0.22–2.59, *p* = 0.656)	0.49 (0.14–1.77, *p* = 0.277)
ICM	0	43 (26.5)	-	-
	1	119 (73.5)	1.33 (0.44–4.02, *p* = 0.610)	2.60 (0.81–8.37, *p* = 0.110)
DCM	0	119 (73.5)	-	-
	1	43 (26.5)	0.75 (0.25–2.26, *p* = 0.610)	NA (NA-NA, *p* = NA)
Kidney disease	0	107 (66.0)	-	-
	1	55 (34.0)	1.73 (0.70–4.26, *p* = 0.234)	0.92 (0.31–2.70, *p* = 0.883)
Age	Mean (SD)	61.4 (9.2)	1.03 (0.97–1.08, *p* = 0.328)	1.01 (0.96–1.07, *p* = 0.639)
Preop creatinine mg/dL	Mean (SD)	1.4 (0.8)	1.24 (0.89–1.73, *p* = 0.206)	1.22 (0.67–2.25, *p* = 0.515)

SR: sinus arrhythmia; pAF: paroxysmal atrial fibrillation; peAF: permanent atrial fibrillation; ICM: ischemic cardiomyopathy; DCM: dilated cardiomyopathy

**Table 5:** Linear regression revealed that right heart failure was a dependent factor

Model Coefficients – RHF							
						95% Confidence Interval Lower upper	
Predictor	Estimate	SE	Z	p	Odds ratio		
Intercept	-4.22	**2.10**	**-2.01**	**0.045**	**0.01**	**0.00**	**0.90**
ICM	2.86	**1.15**	**2.49**	**0.053**	**17.45**	**1.84**	**33.41**
DCM	-	**-**	**-**	**-**	**-**	**-**	**-**
Kidney disease	0.13	**0.63**	**0.20**	**0.838**	**1.14**	**NA**	**NA**
Preop creatinine mg/dL	0.47	**0.34**	**1.37**	**0.170**	**1.59**	**0.33**	**3.89**
Age	-0.02	**0.03**	**-0.84**	**0.403**	**0.98**	**0.82**	**3.12**
Female	-1.30	**0.88**	**-1.47**	**0.142**	**0.27**	**0.92**	**1.03**
Groups							
pAF – SR	1.04	**0.69**	**1.49**	**0.135**	**2.82**	**0.05**	**1.54**
peAF – SR	2.54	**0.84**	**3.02**	**0.003**	**11.02**	**0.72**	**12.84**

SE: standard error; Z: Z test; SR: sinus arrhythmia; pAF: paroxysmal atrial fibrillation; peAF: permanent atrial fibrillation; ICM: ischemic cardiomyopathy; DCM: dilated cardiomyopathy

### Inverse probability weighting and the estimated average treatment effect

After performing IPTW, we estimated the ATE using SR, pAF and peAF as the treatments and post-LVAD IS as the outcome and found that patients with either pAF or peAF had a significantly greater possibility of developing IS post-LVAD implantation (Table 6). Compared with patients with SR, those with pAF had a 15% increased risk of post-LVAD IS, and those with peAF had a 36% increased possibility of developing IS post-LVAD implantation (*p* = 0.035 and *p* = 0.004) (Table 6). We also calculated the ATE for mortality after LVAD implantation, and found that compared with those with SR, patients with peAF had a 54% increased risk of mortality (*p* < 0.001). The ATE for post-LVAD RHF did not significantly differ among the three groups (Table 6).

**Table 6:** Average treatment effect after inverse probability weighting

Post-LVAD mortality					
ATE	Coef.	Robust Std. Err.	*P*	95% Confidence interval	
Groups				Upper	Lower
pAF vs. SR	0.08	0.099	0.384	-0.11	0.28
peAF vs. SR	0.54	0.068	< 0.001	0.41	0.68

Post-LVAD ischemic stroke					
ATE	Coef.	Robust Std. Err.	*P*	95% Confidence interval	
Groups				Upper	Lower
pAF vs. SR	0.15	0.07	0.035	0.01	0.30
peAF vs. SR	0.36	0.12	0.004	0.11	0.60

Post-LVAD right heart failure					
ATE	Coef.	Robust Std. Err.	*P*	95% Confidence interval	
Groups				Upper	Lower
pAF vs. SR	0.02	0.06	0.730	-0.10	0.15
peAF vs. SR	0.01	0.11	0.881	-0.19	0.22

pAF: paroxysmal atrial fibrillation; peAF: permanent atrial fibrillation; SR: sinus arrhythmia; Std. Err.: standard error

## Discussion

AF is a well-established risk factor for cardiovascular events in the general population [13]. In this investigation, we examined whether preoperative AF affects survival and adverse events during LVAD therapy.

The results of our investigation may be summarized into three main conclusions. Initially, patients with preoperative peAF who underwent LVAD implantation had an increased likelihood of mortality compared with patients with SR or pAF who underwent LVAD implantation. Furthermore, pAF and peAF were identified as distinct factors that independently increase the risk of postoperative IS. Although patients with either pAF or peAF had higher INR values than patients with SR, we did not detect an increased risk of bleeding events in those patients.

Due to the absence of a physiological pulse, patients with a continuous flow (CF)-LVAD experience extremely elevated levels of sympathetic nerve activity [14] as a consequence of baroreceptor unloading [15]. This may contribute to overt hypertension and difficulty in controlling blood pressure due to the increased total peripheral resistance through the α1 receptor [16, 17]. Endothelial dysfunction is a cellular consequence of CF-LVADs that develops immediately following implantation, progressively worsens, and is linked to deleterious cardiovascular events in this population [17, 18].

The direct contact between blood and the foreign material of an LVAD results in a hypercoagulable state, as well as an increase in inflammatory markers that can promote altered coagulation profiles and predispose patients to platelet activation [19, 20].

Although some minor deviations occur among institutions due to variations in philosophy and practices, patients are typically prescribed warfarin anticoagulation therapy with international normalized ratio (INR) objectives of 2.0–3.0 in addition to antiplatelet therapy [20]. Some of these minor differences are related to the approach used for the cessation of anticoagulation therapy during an episode of gastrointestinal bleeding [20]. These differences include the following: (1) active reversal of the INR upon admission for gastrointestinal bleeding versus observation and simple cessation of anticoagulation; (2) discontinuation of antiplatelet medications versus continuation of either aspirin or a short-term alternative such as a glycoprotein IIb/IIIa inhibitor; and (3) timing of anticoagulation resumption after treatment for GI bleeding [20].

Although no universally applicable approach has been established, numerous authors have reported a low risk of thromboembolic events (TEs) with mild anticoagulation, and most TE events occur at an INR of less than 1.5 [20, 21, 22]. Additionally, the complete cessation of warfarin therapy for an extended period without any adverse events has been documented [23].

Regrettably, no rigorous clinical trials that would inform treatment algorithms have been conducted, and thus the most effective anticoagulation strategy remains to be determined. Intuitively, the same approach may not be appropriate for patients with distinct clinical characteristics [20]. The function of AF in this context has not been the subject of any prior research [20].

The precise mechanism by which AF increases the risk of TE events originating in the left atrium is not completely understood. Recent research has shown that platelet activation and thrombin production occur within the left atrium [24].

It is undisputed that AF is a prothrombotic condition, and the intensity of this state is undoubtedly exacerbated by the LVAD implant. Consequently, it may be necessary to implement modified anticoagulation strategies. In the present era, we have devoted more attention to the left atrial appendage in the context of patients with preoperative AF from a surgical perspective [25]. Currently, we remove the left atrial appendage from patients who have a thrombus in this location, as evidenced by preoperative echocardiography. In this series, this step was necessary in only nine patients. Although only a few existing studies support this practice, it may be beneficial to prophylactically remove the left atrial appendage in patients with preoperative AF to reduce the risk of stroke [20, 25].

Although the most prevalent regimen for patients following LVAD implantation is warfarin anticoagulation and aspirin therapy, no significant clinical trials have examined alternative anticoagulants in this population. In contrast, the efficacy and favorable adverse effect profiles of newer anticoagulants have been demonstrated to be preferable to those of warfarin in patients with AF in three landmark trials [20].

The risk of thromboembolic events is a significant concern among patients with an LVAD, and conflicting clinical data exist regarding whether AF elevates this risk. Although preoperative AF has been linked to an increased risk of thromboembolism [20], other studies have reported a reduced association or no increased risk [8, 9, 10].

A significant finding of our study is that patients with pAF or peAF experienced IS at markedly higher INR values than did those with SR, which is consistent with the findings of Enriquez et al. [34]. Interestingly, although patients with pAF or peAF had higher INR values than did patients with SR, we did not detect an increased incidence of bleeding events, similar to the findings of Enriquez et al. [34]. This finding supports the notion that acquired von Willebrand disease (aVWd) significantly contributes to bleeding in patients with LVADs and that a minor variation in the target INR may be less impactful [35]. In our study, we found that both pAF and peAF are independent risk factors for postoperative IS. This aligns with a previous study that investigated thromboembolism and AF in patients with continuous-flow LVADs, where Stulak et al. [20] reported a heightened risk of thromboembolism associated with preoperative AF (HR: 1.89; *p* < 0.01). The precise mechanism by which AF increases the risk of TE events in the left atrium (LA) remains inadequately understood. AF meets the conditions of Virchow’s triad, which is essential for thrombus formation: blood stasis, endothelial dysfunction, and activation of the coagulation cascade [36]. Endothelial dysfunction is an additional pertinent element of Virchow’s triad that has been identified in patients with AF through the measurement of various markers of endothelial perturbation, including von Willebrand factor and E-selectin [37]. Clotting activation, which represents the third component of Virchow’s triad, potentially contributes to thrombosis-related clinical events in patients with atrial fibrillation [38]. Numerous studies indicate that AF can lead to a hypercoagulable state, as evidenced by elevated plasma levels of F1 + 2, D-dimer, and fibrinogen [39, 40]. Platelet activation and thrombin generation have been shown to occur within the left atrium [41]. AF undeniably signifies a prothrombotic condition, which is unequivocally exacerbated with VAD implantation. Consequently, modified anticoagulation methods may be necessary. From a surgical perspective, increased focus on the LAA has been observed in the contemporary management of patients with preoperative AF.

The connection between AF and HF has long been established. Through decreases in the diastolic filling time, reductions in ventricular filling through the loss of atrial systole, and impacts on systolic function, AF can exacerbate the symptoms of HF [26, 27, 28]. On the contrary, decompensated HF can exacerbate AF and cause ventricular tachycardia. Given this interaction, it is not unexpected that AF has been linked to higher hospitalization and mortality rates in patients with HF [27, 28, 29]. The findings of our study closely align with those of the SOLVD study [27]. In our study, only patients with peAF had significantly lower survival rates than did patients with preoperative SR and patients with pAF according to the IPTW analysis. Our findings regarding poorer survival of patients with preoperative AF are in accordance with those of a large meta-analysis performed by Tantrachoti et al. [30], who analyzed seven retrospective investigations that included 5823 patients with LVADs (AF 1589; SR 4234). The median follow-up duration was between seven and twenty-four months. Pooled analysis revealed a significantly greater mortality risk in patients with preoperative AF who underwent LVAD implantation than in those with SR (risk ratio [RR] 1.16, 95% CI 1.05-1.28; I2 = 0%) [30]. The results of previous studies are conflicting; Antonides et al. [7] used data from a large European multicenter registry to clarify the impact of preoperative AF on outcomes during LVAD support. Antonides et al. [7] reported that patients with AF had a noticeably poorer survival rate than did those with SR; however, the presence of preoperative AF was not significantly independently associated with lower survival in the multivariable model of this investigation.

LVAD management may be affected by our findings in that patients benefit from more aggressive rhythm management to reduce the AF burden. Surgical CryoMAZE, liberal antiarrhythmic medication use, and catheter ablation during LVAD placement might be considered.

While the medical management of AF in the non-LVAD population is well documented, data on the optimal management of AF following LVAD implantation are lacking [31]. Specifically, the influence of AF on hemodynamics and symptoms in patients with a CF-LVAD is not well understood. Consequently, ascertaining the advantages of any specific treatment is difficult [31]. In this context, the medical management of AF in patients with an LVAD is based on either the extrapolation of preestablished guidelines in non-LVAD populations or on limited retrospective or single-center reports [31].

Anticoagulation is necessary for patients with LVADs to mitigate the risk of pump thrombus and TEs, provided that contraindications are not present [32]. The INR target in these patients is generally not affected by AF [31], and rate control is a critical management strategy for these patients [31]. Although the reverse remodeling effects of β-blockers in patients with HF with reduced ejection fraction (HFrEF) [31, 33] do not extend to those with LVADs, 1 of the 3 β-blockers approved for patients with heart failure with a reduced ejection fraction (carvedilol, bisoprolol, or metoprolol succinate) [34] is generally continued in the absence of any clear contraindication (e.g., hypotension, orthostasis, severe RV dysfunction). Perhaps the most frequently employed agent for this purpose, β-blockers can be used to achieve rate control in patients with AF and an LVAD [31]. Digoxin is frequently incorporated into medical management strategies for patients with HFrEF after LVAD implantation and is frequently used as an adjunct to goal-directed medical therapy to reduce the rate of HF-related hospitalizations [35]. The combination of digoxin and β-blockers has been demonstrated to be an effective strategy for achieving AF rate control at rest and during exercise [36]. This strategy may also be considered for patients with AF and an LVAD [31]. The optimal heart rate or rate control strategy for LVAD recipients is unclear and may differ from that of patients without LVADs due to numerous physiological differences [31].

### Study limitations

Our study has multiple limitations. First, the data in this study are constrained by documentation in patients’ medical charts because of its retrospective and nonrandomized design, and thus the study is susceptible to selection bias and confounding factors. This study also has a limited sample size and a small number of patients and outcomes. Therefore, the results should be viewed as preliminary and should serve only to generate hypotheses. 68% of the patients in our study received HMII implants, which accounted for only 31%, and all patients had international normalized ratio (INR) goals based on our institutional protocol. Therefore, our findings of bleeding and thromboembolic events may not be applicable to different INR protocols or other devices. Furthermore, our study yielded significantly favorable results when a subgroup analysis was conducted. Therefore, the findings are less meaningful.

## Conclusions

Preoperative peAF is associated with poorer survival compared with SR after LVAD implantation. Both pAF and peAF are independent risk factors for a higher incidence of IS post-LVAD.

## Data Availability

The anonymized data collected are available as open data in Figshare with the identifier https://doi.org/10.6084/m9.figshare.27796389.

## Abbreviations

AF: Atrial fibrillation
ALT: Alanine aminotransferase
AST: Aspartate aminotransferase
BMI: Body mass index
BSA: Body surface area
COPD: Chronic obstructive pulmonary disease
CVD: Cerebrovascular disease
DCM: Dilated cardiomyopathy
DM: Diabetes mellitus
eCV: Electric cardioversion
EF: Ejection fraction
Hb: Hemoglobin
HR: Hazard ratio
ICM: Ischemic cardiomyopathy
ICU: Intensive care unit
INR: International normalized ratio
INTERMACS: Interagency Registry for Mechanically Assisted Circulatory Support
KD: Kidney disease
LAA: Left atrium appendage
LDH: Lactate dehydrogenase
NT-pro-BNP: N-terminal pro-brain natriuretic peptide
OR: Odds ratio
PAD: Peripheral arterial disease
pAF: Paroxysmal atrial fibrillation
PCI: Percutaneous coronary intervention
peAF: Permanent atrial fibrillation
PH: Pulmonary hypertension
RHF: Right heart failure
SR: Sinus arrhythmia
STS-Intermacs: Society of Thoracic Surgeon Interagency Registry of Mechanically Assisted Circulatory Support
